# Uniaxial Cyclic Tensile Stretching at 8% Strain Exclusively Promotes Tenogenic Differentiation of Human Bone Marrow-Derived Mesenchymal Stromal Cells

**DOI:** 10.1155/2019/9723025

**Published:** 2019-02-21

**Authors:** Hui Yin Nam, Belinda Pingguan-Murphy, Azlina Amir Abbas, Azhar Mahmood Merican, Tunku Kamarul

**Affiliations:** ^1^Tissue Engineering Group, Department of Orthopaedic Surgery (NOCERAL), Faculty of Medicine, University of Malaya, 50603 Kuala Lumpur, Malaysia; ^2^Department of Biomedical Engineering, Faculty of Engineering, University of Malaya, 50603 Kuala Lumpur, Malaysia

## Abstract

The present study was conducted to establish the amount of mechanical strain (uniaxial cyclic stretching) required to provide optimal tenogenic differentiation expression in human mesenchymal stromal cells (hMSCs) *in vitro*, in view of its potential application for tendon maintenance and regeneration. *Methods*. In the present study, hMSCs were subjected to 1 Hz uniaxial cyclic stretching for 6, 24, 48, and 72 hours; and were compared to unstretched cells. Changes in cell morphology were observed under light and atomic force microscopy. The tenogenic, osteogenic, adipogenic, and chondrogenic differentiation potential of hMSCs were evaluated using biochemical assays, extracellular matrix expressions, and selected mesenchyme gene expression markers; and were compared to primary tenocytes. *Results*. Cells subjected to loading displayed cytoskeletal coarsening, longer actin stress fiber, and higher cell stiffness as early as 6 hours. At 8% and 12% strains, an increase in collagen I, collagen III, fibronectin, and N-cadherin production was observed. Tenogenic gene expressions were highly expressed (*p* < 0.05) at 8% (highest) and 12%, both comparable to tenocytes. In contrast, the osteoblastic, chondrogenic, and adipogenic marker genes appeared to be downregulated. *Conclusion*. Our study suggests that mechanical loading at 8% strain and 1 Hz provides exclusive tenogenic differentiation; and produced comparable protein and gene expression to primary tenocytes.

## 1. Introduction

Bone marrow-derived mesenchymal stromal cells (MSCs) have the ability to undergo multilineage differentiation and, when introduced into damaged tendon, have been shown to result in superior repair outcomes [1, 2]. Despite demonstrating good efficacy, there have been concerns that undifferentiated cells may possibly progress towards unwanted cell lineages when transplanted into tissues, resulting in patient morbidity [3, 4]. An example to demonstrate such phenomenon would be in the formation of osteoblastic cells when human MSCs (hMSCs) are transplanted into the cartilage tissue [5]. It has been suggested that lineage-committed or predifferentiated hMSCs may be the answer to this problem [6]. Several methods can be employed to direct hMSCs towards a particular lineage. In the past, these have included hormonal, ionic, and environmental manipulation [7]. However, one of the mechanisms that can be readily used on these cells but not often described in literature is mechanical signalling [8].

It is suggested that the ability of cells to respond to mechanical stimuli is controlled by a series of mechanosensitive receptors or structures that sense and convert mechanical signals into biochemical signalling events [9]. This process, commonly known as mechanotransduction, translates mechanical cues that are perceived from the environment into intracellular signals. This ultimately regulates the complex processes involved in cell proliferation and differentiation [10]. It has been described that during this process, the complex interaction of signals generated from the binding of integrins to signalling molecules, the opening of stretch sensitive ion channels, and the resultant cytoskeletal deformation are simultaneously activated [11]. However, the order of sequence of these events, as well as the relationship between the activated pathways and outcome, remains to be rationalized [12, 13].

Although previous works have indicated that mechanical stimulation in general guides MSC differentiation in different ways, these studies have predominantly involved cells other than those found responsible for tendon or ligament homeostasis, such as osteoblast, neuron-like cells, and chondrogenic cells [14–16]. In addition, there appears to be very few studies investigating the effects of cyclic uniaxial tensile loading on progenitor cells, although this stimulus is physiologically relevant to the musculoskeletal system. It is worth noting that this stimulus is probably the single most important signal that regulates the proliferation and functions of both ligament and tendon cells [17, 18]. However, we also need to be mindful that because of their multipotential ability, it is possible that stimulating stem cells mechanically at an inappropriate manner can result in undesirable outcomes as previously mentioned. It is therefore paramount that the characteristics of the mechanical loading applied be established so as to eliminate these unwanted outcomes. Sadly, studies related to this area appears lacking as previous studies have been mainly focused on a narrow range of frequency and strain rates which do to mimic the scenario observed during physiological loading [19–22].

In order to establish these characteristics, the present study was conducted to examine the effects of uniaxial cyclic stretching in different durations and strain rates on hMSCs. The focus of this study is to determine the mesenchymal lineage differentiation potential of these cells using gene expression and extracellular matrix (ECM) production related to mesenchymal lineage-related specific markers. These were also compared to tenocytes in order to determine if the tenogenic expression potential of hMSCs subjected to these loading conditions was comparable to that of native tendon cells. These would then indicate that progenitor cells would have undergone tenogenic differentiation. Tenogenic differentiation is defined as cells that exhibit tenocyte-lineage marker genes at both mRNA and protein levels [23]. Amongst the genes that have been identified as described in many literatures include scleraxis, tenomodulin, tenascin-C, collagen type I, collagen type III, and decorin [19, 23–25]. Protein expressions for tenogenic differentiation on the other hand are less specific and not well described. However, from available literature, frequently quoted proteins that appear to be relevant to the tenogenic differentiation process have included collagen I and collagen III [23, 26].

We hypothesize also that the regulation of extracellular matrix remodelling as well as the expression of the differentiation of hMSCs to a particular cell lineage is dependent on the degree of tensile forces; the morphology and stiffness of the cells were also investigated. Since the focus of this study is relating to the tenogenic differentiation potential of cyclic-loaded hMSCs, the expression of tenogenic genes and proteins as mentioned above was thus investigated. It is hoped that by determining the effects of mechanical stretch on hMSCs using quantitative measurements, we may be able to have better understanding on the mechanical characteristics that govern tendon homeostasis thus enabling future potential therapies for tendons and ligaments to be advocated.

## 2. Materials and Methods

All experimental protocols were approved by the University Malaya Medical Centre Institutional Review Board (Reference no: 369.19) and performed in accordance with the guidelines for Medical Ethics Committee of the University Malaya Medical Centre.

### 2.1. Isolation and Culture of Human Bone Marrow-Derived MSCs

To isolate bone marrow-derived MSCs, the bone marrow was aspirated from the femoral canal of 10 patients/donors undergoing orthopaedic-related surgeries such as total joint arthroplasty in the University Malaya Medical Centre. Each bone marrow sample was kept on ice throughout the transportation to the laboratory and processed for cell isolation as described in our previous publication [27]. The cells were subcultured until passage 2 to be used in our experiments.

**Figure 1:**
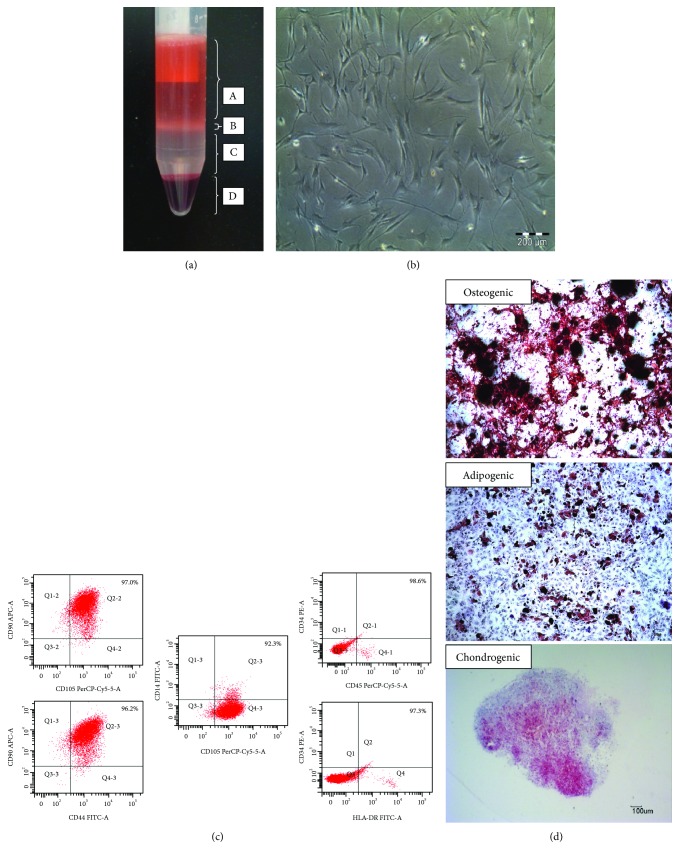
Characterization of hMSCs was confirmed. (a) Density gradient separation of human bone marrow: (A) plasma, (B) mononuclear cells, (C) ficoll paque, (D) erythrocytes, and granulocytes. (b) Morphology of replated cells showed homogeneous and fibroblastic shape. (c) Representative images of multicolour CD markers by flow cytometry. The results showed that hMSCs expressed at least 90% of double-positive expression, double-negative, or coexpressed positive and negative markers. (d) Trilineage differentiation potential of the hMSCs into osteogenic, adipogenic, and chondrogenic lineage.

To determine whether the cells obtained were hMSCs, various tests including flow cytometry analysis for specific cell surface markers, cell morphological images, and the ability of the isolated cells to undergo trilineage differentiation were conducted. The methods used in this study are described in our previous publications [27, 28]. The isolated cells appeared to conform to the characteristics expected of MSCs (Figure 1), i.e., (1) spindle-shaped plastic adherent features; (2) positive markers for CD29, CD44, CD73, CD90, and CD105 while being devoid of CD14, CD34, CD45, and HLA-DR [28]; and (3) able to undergo trilineage differentiation, namely, chondrogenic, osteogenic, and adipogenic differentiation.

### 2.2. Isolation and Culture of Human Tenocytes

Human primary tenocytes were isolated from hamstring tendons of adult donors, who underwent surgery for joint arthroplasty. Tendon tissues were harvested to the required size by the operating surgeon and transferred aseptically into containers and immersed with saline solution. Once the tendons were harvested, cell isolation was immediately performed, using the methods modified from the study of Zhang and Wang [29]. Briefly, the tendons were minced into approximately 1 mm^3^ in size under a sterile condition, and then phosphate buffered saline (PBS) was added (Gibco, USA). Subsequently, the mixture was added with 0.4 mg/mL type I collagenase and incubated at 37°C for 2 h to allow the enzymatic digestion process to occur. After digestion, the suspension was centrifuged at 1800 rpm for 5 min to remove the collagenase solution, then the pellet was washed 2 times with PBS by centrifugation. The pellet was then resuspended with 1 mL of DMEM high glucose (4.5 g/L glucose) supplemented with 10% fetal bovine serum (FBS), 1% penicillin-streptomycin, and 1% GlutaMAX™-I (Gibco, USA), and transferred into a T25 flask which was added with 5 mL of culture medium. Cultures were incubated at 37°C and 5% CO_2_ incubator for 24 h. The digested tissues were then removed from the cell culture flask and discarded completely. The culture medium was changed every third day, until 80–85% confluency for subculture using trypsin digestion. These primary native human tenocyte cultures (passage 3) were used as positive controls in the subsequent experiments.

### 2.3. Application of Cyclic Uniaxial Tensile Strain

A commercial loading device (STREX, Japan) fitted with elastic silicone chambers was used to conduct experiments that determine the effect of cyclic uniaxial strain on hMSCs. hMSCs were seeded on the collagen type I (Sigma, USA)-coated silicone chamber at the density of 10^4^/cm^2^ and allowed to set at 37°C in complete growth medium for 48 h. The medium was then changed to 1% FBS for 24 h and proceeded with complete growth medium before being assembled into the uniaxial strain device. Control cells were treated similarly but were not subjected to cyclic stimulation. The medium and cells were harvested after 6, 24, 48, and 72 h of cyclic loading for downstream analyses, which included (1) biochemical assay, (2) immunostaining, (3) immunophenotyping, (4) topography imaging and elasticity measurement, and (5) gene expression assay.

### 2.4. Cellular Morphology by Microscopy

Phase-contrast microscopic images of unstrained and strained hMSCs were obtained (Olympus, Japan) in at least four randomly selected sites from our visual field. To observe the effect of cyclic loading on cytoskeletal actin arrangements, hMSCs at all conditions were stained with fluorescent phallotoxins (Molecular Probes, Oregon, USA) for 30 min and then the nucleus stained with Hoechst (Molecular Probes, Oregon, USA) for 10 min in the dark. Fluorescence was recorded using a laser scanning confocal attachment (Leica TCS SP5 II, Germany) and measured by LAS AF image software (Leica, Germany). Images of unstrained MSCs on silicone membrane served as control.

### 2.5. Quantification of ECM Components

At the end of each time point of the experiment, the total amount of collagen, sulfated glycosaminoglycan (sGAG), and elastin of the resulting samples was determined using Sircol™ Collagen assay kit, Blyscan™ sGAG assay kit, and Fastin™ Elastin assay kit, respectively. The technique used in each measurement was according to the manufacturer's (Biocolor, UK) protocol. These kits used quantitative dye-binding methods to determine the total quantity of the respective ECM component in the sample which released to medium. An enzyme immunoassay kit (Chondrex Inc., USA) was used to measure the levels of type I collagen in strained hMSC lysate (1 Hz, 8%) following the manufacturer's instructions. The concentration of collagen type I was obtained by measuring the absorbance at 490 nm on the microplate reader.

### 2.6. Immunocytochemical and Fluorescent Immunostaining for ECM Analysis

Membranes with hMSCs subjected to the uniaxial straining or in unstrained conditions were rinsed using PBS, followed by fixation process in methanol for 20 min. After rinsing using Tris-buffered saline (Dako, Denmark), peroxidase block was applied for 5 min to reduce nonspecific background signalling. Cells were then incubated with primary antibodies, which included rabbit anti-collagen type I, rabbit anti-collagen type II, or rat anti-collagen type III (Calbiochem-Daiichi Fine Chemical Co., Japan) diluted at 1 : 100 for 30 min. The cells were then incubated with streptavidin-peroxidase secondary antibody (Dako, Denmark) for 30 min. At last, the collagens in the cells were visualized by reaction with diaminobenzidine (Dako, Denmark).

For direct visualization of the adhesion molecules fibronectin matrix and N-cadherin, 4% paraformaldehyde was used to fixed cells and was permeabilized with −20°C acetone. Cells were then incubated with 1% bovine serum albumin to block nonspecific binding of antibodies, before being incubated with primary antibody, anti-fibronectin (Abcam, UK) diluted 1 : 300 for 1 h. The primary antibody was then detected by a secondary antibody specific to rabbit IgG (Abcam, UK) diluted 1 : 600 for 1 h. Hoechst staining was performed at the end of the staining process and examined under laser scanning confocal microscope (Leica TCS SP5 II, Germany).

### 2.7. Stimulated Cell Surface Antigen Analysis by a Fluorescence-Activated Cell Sorter (FACS)

Antibodies against the human antigen, CD44, CD73, CD90, and CD105 (BD Biosciences, USA), were used to characterize the surface antigen expressions of stretched hMSCs. Briefly, the loaded cells were resuspended in 100 *μ*L of PBS and incubated with fluorescein isothiocyanate- (FITC-) or phycoerythrin- (PE-) conjugated antibodies in the dark for 15 min at room temperature. The fluorescence intensity of the cells was evaluated using a flow cytometer (BD FACS Cantor II, BD Biosciences, USA). Data were analysed using CELLQUEST software (BD Sciences, USA). The presence or absence of staining in cells was determined by comparing strained cells to the matched unstrained control.

### 2.8. Histologic Assessment of Differentiation after Mechanical Stimulation

The presence of bone-forming nodules was used to determine the occurrence of osteoblast differentiation. This was further assessed using Alizarin Red S dye (Sigma, USA), which stains calcium phosphate deposits. The accumulation of lipid droplets was used to denote adipocyte differentiation. It was determined by incubating paraformaldehyde-fixed cells with 60% isopropanol and followed by freshly prepared Oil Red O solution (Sigma, USA). Unstrained samples were treated as controls. All samples were then captured using a light microscope (Nikon Eclipse TE2000-S, Japan).

### 2.9. Atomic Force Microscopy Measurement of Young's Modulus

Atomic force microscopy (AFM) images were obtained by scanning the cell surface under ambient conditions using AFM (Bruker Nano, USA) that was set at PeakForce QNM mode. The AFM measurements were obtained using ScanAsyst-air probes. However, the spring constant (nominal 0.4 N/m) and deflection sensitivity were first calibrated but not the tip radius (the nominal value has been used, 3.5 nm). AFM images were collected from each sample and at random spot (at least five areas per sample). The quantitative mechanical data was obtained by measuring DMT modulus/Pa using Bruker software (NanoScope Analysis). To obtain Young's modulus, the retracted curve was fit using the Derjaguin-Muller-Toporov model or abbreviated as DMT modulus [30].

### 2.10. Multiplex Gene Expression Assay

Total RNA was extracted from unstrained and strained hMSCs using RNeasy mini kit (Qiagen, USA). The purity and concentration of the RNA were assessed by determining the absorbance ratio, measured at 260 and 280 nm wave bands. RNA integrity was assessed by visualizing 18S and 28S rRNA bands on formaldehyde-agarose gels. Only samples with high quality were selected for microsphere-based multiplex-branched DNA downstream analysis. The mRNA expression of mesenchymal lineages (Table 1) was quantified by the QuantiGene 2.0 Plex assay (Panomics/Affymetrix Inc., USA). Individual bead-based oligonucleotide probe sets specific for each gene examined were developed by the manufacturer (the 2.0 plex set 12082). In this assay, each lysate was measured in triplicate wells. Controls are also included for genomic DNA contamination, RNA quality, and general assay performance. The housekeeping gene was *PGK1* (phosphoglycerate kinase 1) previously validated as the best housekeeping for accurate gene expression analysis in our study.

### 2.11. Statistical Analysis

The assays were carried out with a minimum number of technical triplicates (*n* = 3) per experimental run, using six independent samples from different donors (*N* = 6) for each group of the experiments. Data were presented as mean ±  standard deviation (SD). For Young's modulus experiment, Student's *t*-test was carried out to compare the differences in mean values. While for the other experiments, statistical significance was analysed by one-way analysis of variance (ANOVA), using the least significant difference (LSD). A confidence level of 95% (*p* < 0.05) was chosen for determining statistical significance using the SPSS 15.0 software (SPSS Inc., USA).

## 3. Results

### 3.1. Uniaxial Mechanical Strain Induces MSC Alignment Perpendicular to the Direction of Stretching

**Figure 2:**
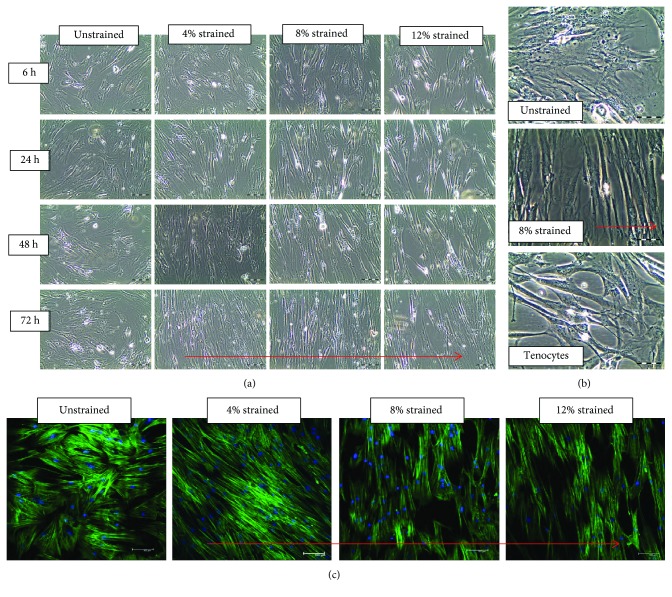
Microscopy images of unstrained and strained hMSCs. (a) Phase-contrast photomicrographs of hMSCs subjected to cyclic uniaxial stretching in different magnitude and duration of stretching. (b) Higher magnification of phase contrast of unstrained and 8% strained hMSCs at 72 h and tenocytes. (c) Confocal laser scanning micrographs showing actin stress fibers (green) and nuclei (blue) of unstrained cells and 4%, 8%, and 12% strained cells at 72 h. The substrate was stretched in the red arrow direction.

To determine the effects of uniaxial cyclic strain on cell morphology and organization, hMSCs were exposed to uniaxial strain under predetermined experimental conditions. The degree of cells' responsiveness was affected at different strain magnitude and duration (Figure 2(a)). Cells that were exposed to the highest strain magnitude (12%) aligned themselves faster than cells at other strain rates. After 72 h, cells under cyclic strain aligned themselves perpendicular to the direction of strain and these cells look more elongated and were slender in shape, while unstrained cells remain randomly oriented.

Confocal images showed the reorganization of actin filaments perpendicular to the direction of strain whilst random organization of actin filaments for unstrained cells. It also showed that stained actin filaments were denser in the stimulated hMSCs compared to the nonstimulated groups (Figure 2(c)). hMSCs on 8% uniaxial strained at 1 Hz (Figure 2(b)) lead to spindle-shaped cells similar in shape to tenocytes *in vitro*. All these results indicated that the cellular cytoskeletal development was associated with strain magnitude.

### 3.2. Uniaxial Tensile Loading Enhances Collagen and Elastin Production but Not GAG

**Figure 3:**
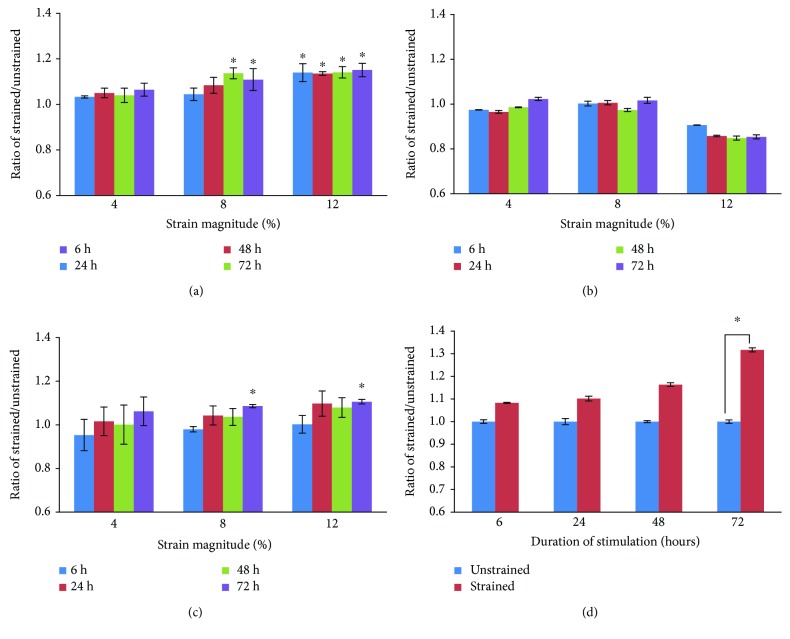
Biochemical analysis of MSCs subjected to various mechanical stimuli for different duration of stimulation. Content of (a) total collagen, (b) GAG, and (c) elastin of strained cells was measured to determine the total quantity of the respective ECM component in the sample which released to medium. (d) The level of collagen type I in the medium was measured by ELISA. The ratio of the ECM expression was counted by normalizing to the expression amount of corresponding unstrained groups (indicated as 1). Significance *p* < 0.05 was represented by ^∗^ which compared to unstrained. *N* = 6, *n* = 3, error bar ± SD.

The total collagen and elastin production appears to be influenced by the strain magnitude. Our results showed that uniaxial stretching increased collagen production (Figure 3(a)), with the exception of the 4% strained group. Higher collagen production was measured as early as 6 h in the 12% strained group. For the 8% strained group, the collagen production was enhanced significantly only after 48 h, which is close to the collagen content in tenoctyes (ratio of human tenoctyes/unstrained hMSCs = 1.43, graph not shown). Compared to collagen, elastin was only increased after 72 h at the higher strained group (Figure 3(c)). However, no enhancement of GAG production in any of the strained groups was observed (Figure 3(b)).

Since collagen type I was reported to be abundant in tendon, ligament, and muscle cells, the 8% strained cells at 1 Hz were further tested using ELISA assay. The results showed that the collagen type I level in medium was increased in mechanically stimulated cells as compared to unstrained cells. The content of collagen type I increased with the duration of stretching (Figure 3(d)).

### 3.3. Mechanical Stimulation Promotes Collagen Type I, Collagen Type III, Fibronectin, and N-Cadherin Expressions

**Figure 4:**
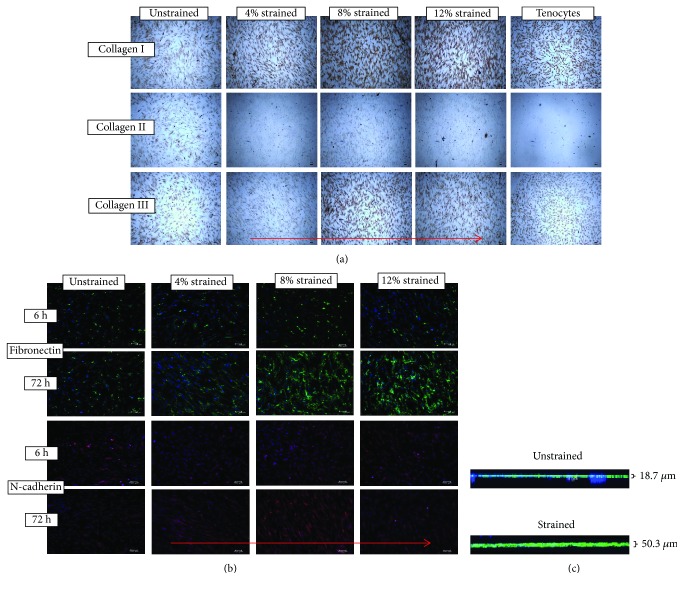
ECM expression on unstrained and strained cells. (a) Comparison of different collagen staining on various mechanical stimuli hMSCs at 72 h and tenocytes as positive control. (b) Immunofluorescence staining of fibronectin and N-cadherin on unstrained and strained hMSC for 6 h or 72 h. The expression of fibronectin and N-cadherin was enhanced by the cyclic stretch and magnitude strain dependent. The substrate was stretched in the red arrow direction. (c) Thicker fibronectin fibrils were formed by cyclic mechanical stimulation.

Immunocytochemical assay showed that the uniaxial cyclic straining promoted the synthesis of collagen type I in MSCs. In the unstrained control group, there was only a light brown collagen staining in the cytoplasm, while a more intense staining was observed in the 72 h strained group for collagen type I (Figure 4(a)). This was in line with the result of collagen type I obtained from ELISA. Collagen I and collagen III staining showed positive protein expression on both unstrained and strained hMSCs but denser in strained cells especially in the 8% and 12% groups. In contrast, collagen II was not expressed when hMSCs were stretched. These results appear comparable to the level of collagen expressed from primary tenocytes.

When unstretched, fibronectin was arranged in random web-like structures, which distributed mainly at the cell periphery. The peripheral fibronectin staining appears to be upregulated when cells are stretched. Fibronectin fibril formation also appears to be enhanced with the increase in strain magnitude (Figure 4(b)). Furthermore, unstimulated or unstretched cells appeared to have thin fibronectin fibrils clustered and distributed throughout the entire basal surface of the cell, while cells exposed to 72 h at 8% and 12% uniaxial stretching appeared to form thicker fibronectin fibrils and to have an observable increase in fibronectin fluorescence intensity (Figure 4(c)). To view cell-cell contacts after stretching, we found that the expression level of N-cadherin was higher on strained cells (Figure 4(b)). However, this level of expression was lower in the 12% strained group.

### 3.4. Mechanical Stretching Induces the Alteration in hMSC Surface Antigen Expression

**Figure 5:**
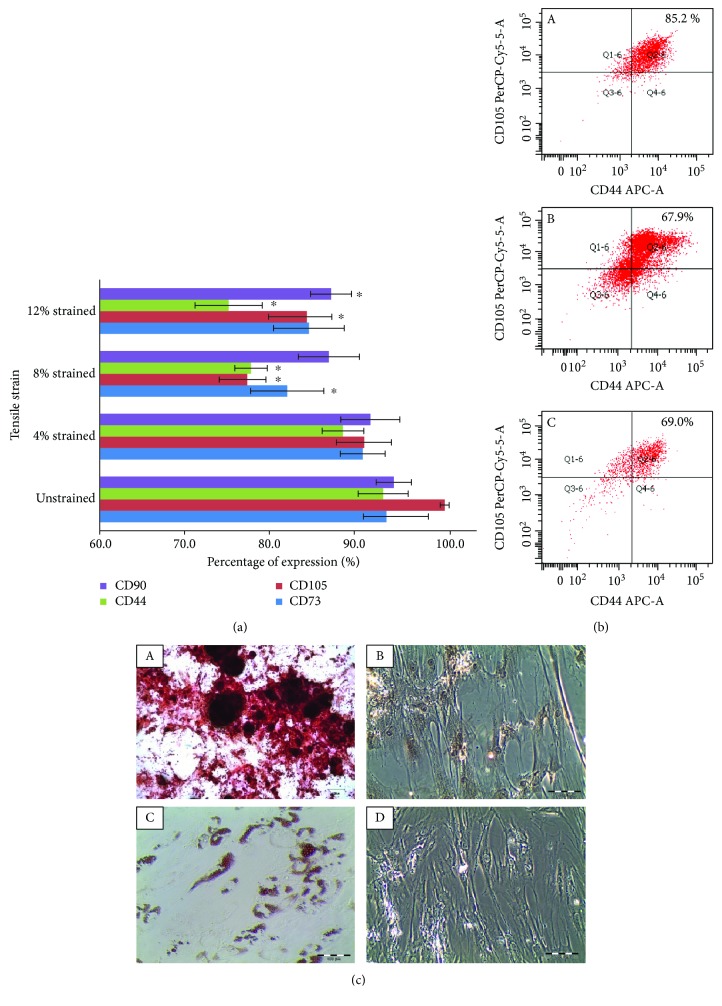
Mechanical stretching altered hMSC surface antigen expression but did not express osteogenesis and adipogenesis. (a) Expression levels of the CD markers of hMSCs cultured in mechanical stretching with different strains. Significance (*p* < 0.05) was represented by ^∗^ which compared to unstrained. *n* = 3, error bar ± SD. (b) Percentage of multicolour expression for lymphocyte adhesion molecule CD44 and endoglin CD105. Fluorescent expression intensity and area of CD44 and CD105 in (A) 4%, (B) 8%, and (C) 12% strain magnitudes. (c) Representative images of Alizarin Red- and Oil Red O-stained hMSCs: (A) positive Alizarin Red staining on osteogenic medium cultured hMSCs, (B) negative Alizarin Red result on mechanical stimulated hMSCs, (C) positive Oil Red O staining on adipogenic medium cultured hMSCs, (D) negative Oil Red O result on strained hMSCs.

The expression of the CD markers in hMSCs appears positive in nonstimulated cells on silicone chambers, as with hMSCs cultured on plastic culture flasks. After 72 h of cyclic loading, CD markers in 4% strained cells appear comparable to unstrained cells. However, when subjected to 8% and 12% strains, the expression of CD markers was reduced, suggesting that appropriate levels of mechanical stretch may induce the alterations in MSC surface antigen expression (Figure 5(a)). It was observed that CD44 and CD105 were significantly reduced in both 8% and 12% strained groups, while CD73 and CD90 reduced significantly at 8% and 12% strains, respectively.  Figure 5(b) demonstrates examples of the expression of the multicolour CD markers in different strain magnitude.

### 3.5. Mechanical Stretching Did Not Express Osteogenesis and Adipogenesis Histological Staining

Mineralization of hMSCs was observed in the osteogenic medium induction hMSCs (21 days) after being stained with Alizarin Red S (Figure 5(c)A), but this was not shown in mechanical induction hMSCs (8% strained, 72 h), with only slightly brown stained around the nucleus of the cells (Figure 5(c)B). Negative results were shown in 4% and 12% strained cells (figure not shown). The effect of tensile loading on adipogenic differentiation of hMSCs was studied using Oil Red O staining of the lipid droplets. The lipid droplet formation under adipogenic differentiation was found in adipogenic medium induction hMSCs (14 days) (Figure 5(c)C), whereas the mechanical-stimulated hMSCs showed no lipid droplet (8% strained, 72 h) (Figure 5(c)D). Similar result also appeared on the other 2 strained groups (photo not shown).

### 3.6. Topographical Changes Observed in Mechanically Stimulated Cells

**Figure 6:**
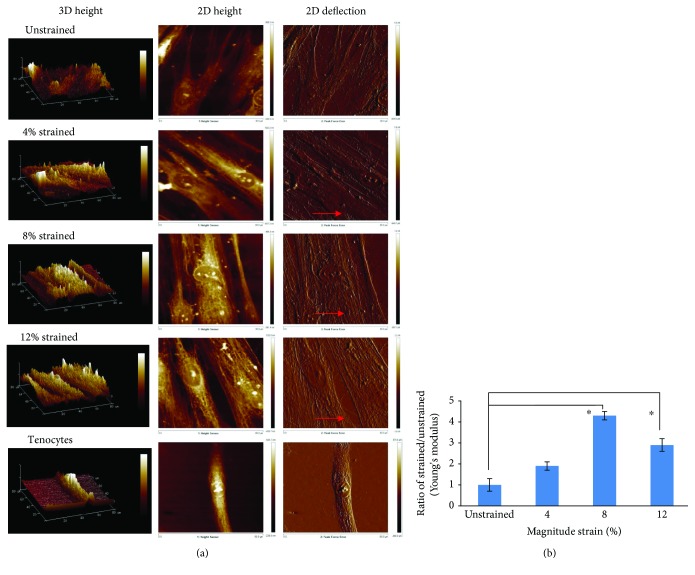
The comparison of cell surface topography between the unstrained hMSCs, strained hMSCs, and tenocytes, visualized by AFM. (a) Representative AFM height and deflection scans of unstrained hMSCs and 4%, 8%, and 12% strained hMSCs, and tenocytes. In height images, brighter colour indicates higher distance of substrate. In deflection images, the detailed structure of presumably the stress fiber could be observed with AFM in different cell groups. The direction of uniaxial strain was in the red arrow direction. (b) Young's modulus on the cytoskeleton of the cells subjected to 4%, 8%, or 12% cyclic stretching for 72 h as indicated. The ratio was counted by normalizing to the expression amount of corresponding unstrained groups (indicated as 1). Statistical significance (*p* < 0.05) was represented by ^∗^ relative to the unstrained group. *n* = 3, error bar ± SD.

The changes in cell topography of unstrained and strained hMSCs were analysed using an AFM. Topographical images were obtained in both height and deflection channels (Figure 6(a)). Results of AFM analysis revealed that strained cells appeared elongated, with spindle-like morphology and microfilament bundles running parallel to their long axes, while unstrained cells appeared large and flat. Height image showed larger height scale for strained cells than unstrained cells. This was apparently related to the thicker actin stress fibers of the strained cells than the unstrained hMSCs, which could be visualized in detail in the deflection channel. In unstrained cells, deflection image revealed the fine cytoskeletal structure (presumably actin) just under the cell membrane at detail. The fine cytoskeleton structure began integrating when mechanical stimulation was applied on the cells. The cytoskeleton of the stimulated cells became more pronounced. This effect was much evident with the higher magnitude strain to hMSCs, compatible with tenocytes.

The elasticity measurements (Young's modulus) were performed on the cytoskeleton regions surrounding the nuclei.  Figure 6(b) shows the average Young's modulus of fixed unstrained and strained hMSCs from 3 independent cultures with 5 different areas. The Young's moduli values of strained hMSC groups were greater than those of unstrained hMSC groups, with a significant increase observed in the 8% and 12% strained group. These results demonstrate that as the strain rate is increased, Young's modulus and therefore stiffness of the cytoskeleton of hMSCs increase. The unstrained hMSCs are supple when compared to strained hMSCs, especially in the 8% strained group.

### 3.7. Mechanical Stimulation Influences the Expression of *MMP3* and *PRR16*

**Figure 7:**
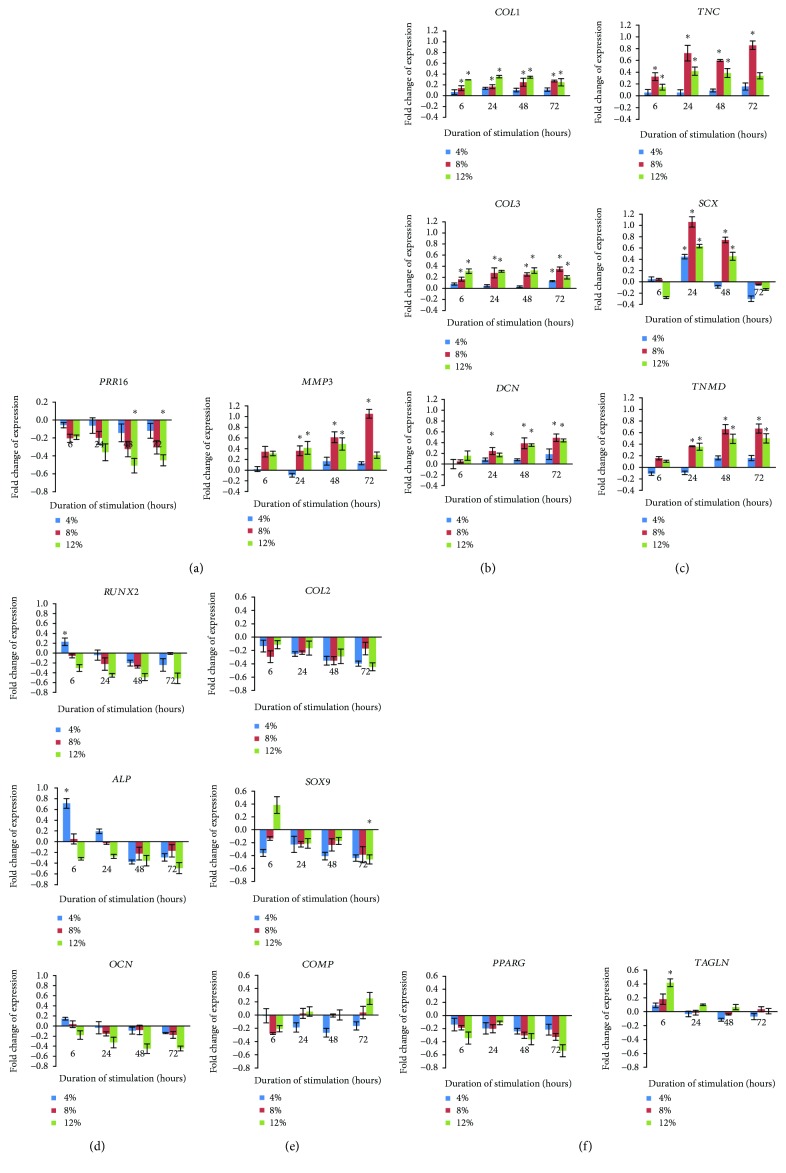
mRNA expression level of different genes subjected to different strain for different time point. (a) mRNA expression of *MMP3* and *PRR16*. (b) ECM component (*COL1*, *COL3*, and *DCN*). (c) Tendon cell lineage (*TNC*, *SCX*, and *TNMD*). (d) Bone cell lineage (*RUNX2*, *ALP*, and *OCN*). (e) Cartilage cell lineage (*COL2*, *SOX9*, and *COMP*). (f) Adipose cell (*PPARG*) and smooth muscle cell (*TAGLN*). The expression level of each gene was normalized with the level of housekeeping gene. The value of fold change was presented as the ratio of strained group with unstrained group. Statistical significance (*p* < 0.05) was represented by ^∗^ which compared to unstrained. *N* = 6, *n* = 3, error bar ± SD.

The mRNA expression of *PRR16*, an indicator of stem cell differentiation, when cells were subjected to mechanical loading is shown in Figure 7(a). At 1 Hz stretching, downregulation of the PRR16 gene was noted in both 8% and 12% strained groups. This effect was more obvious after the cells were stretched for a longer period. These results appear to occur in parallel to the reduction in the expressed CD markers described previously. Although there is a downregulation of *PRR16*, the mRNA expression of *MMP3* was upregulated in 8% strain (Figure 7(a)). The exhibitory effect on *MMP3* mRNA expression was not obvious in the 12% strained group after 48 h.

### 3.8. High Mechanical Strain Upregulated Genes for Macromolecular Components of ECM and Induced Differentiation Markers for Tendon-Like Cell

Uniaxial strain regulated matrix remodelling, as observed from the increasing levels of *COL1* and *COL3* expression, in a liner fashion and parallel to the amount of strain (Figure 7(b)). A significant increase was induced by strains of 8% and 12%, but this upregulation was not significant for the 4% strained group. The expression of *COL3* showed a pattern similar to that of *COL1*, but the increase was slightly higher than that of *COL1* at 8% strain (at 24 h). *DCN* expression was significantly upregulated at 8% and 12% strains (>24 h and 48 h), respectively.

The differentiation of hMSCs towards tendon-like cells was further examined by measuring the expression of several genes (Figure 7(c)). The results demonstrated that the tenogenic marker (*TNC*, *SCX*, and *TNMD*) expression was upregulated in all groups. However, this was only significantly increased in the 8% and 12% strained groups, most notable being in the 8% group after 24 h, i.e., which was closer to the gene expressions from tenoctyes (*DCN* = 1.50, *COL1* = 1.59, *COL3* = 1.37, *TNC* = 2.22, *SCX* = 2.65, and *TNMD* = 1.80; fold change of human tenoctyes vs. unstrained hMSCs; graph not shown). After 2 days of stretching, the gene expression levels of *SCX* returned to the basal level as with the unstrained group, suggesting that the observed increase in gene expression was transient.

### 3.9. Uniaxial Mechanical Strain Did Not Induce Chondrogenic, Adipogenic, and Osteogenic Differentiation Markers

To determine the global differentiation responses of hMSCs when subjected to uniaxial mechanical strain and to ascertain the possible expression of nontendon differentiation markers, the expressions of nontendon genes were also investigated in this study. These included gene markers for the bone, cartilage, and fat. We found that at 4% strain, osteogenic genes (*RUNX2*, *ALP*, and *OCN*) were transiently upregulated (Figure 7(d)). However, at 8% and 12% strains, these genes were downregulated suggesting that at low mechanical strain levels, osteoblastic differentiation is transiently enhanced. Consistent with our immunostaining results, uniaxial strain did not increase *COL2* (Figure 7(e)) and *PPARG* (Figure 7(f)) genes related to chondrogenesis and adipogenesis processes in these progenitor cells. Several molecules involved in chondrogenesis (i.e., *SOX9* and *COMP*) were influenced by the changes in strain magnitude and duration of cyclic stretching (Figure 7(e)). *SOX9* gene was downregulated when uniaxial strain was applied, although at 12% it was observed that a transient increase can be expected at the early stages of stretching (6 h) but is not present thereafter. In contrast, *COMP* was upregulated in the 12% strained group at 72 h. One reason to this may be due to the fact that *COMP* is not a specific gene for chondrogenesis and can be found in tendon cells as per observed in other studies [16]. Uniaxial cyclic stimulation also increased the smooth muscle contractile marker, *TAGLN*, transiently at 12% strain (Figure 7(f)).

Despite the evidences from this study suggesting that a transient increase in nontendon-related genes can occur when hMSCs are subjected to cyclic loading, the functional significance of these changes may be insignificant since only low levels and short duration of these genes were expressed throughout our experiments. We can therefore conclude that uniaxial cyclic loading generally results in tenogenic differentiation and results in the insignificant increase in other downstream musculoskeletal lineages.

## 4. Discussion

Our current study demonstrates that uniaxial stretching over a period of time provides exclusive tenogenic lineage differentiation ability in hMSCs. The genes and proteins expressed from these cells were within the defined characteristics of tenogenic differentiation, as mentioned earlier. We can also conclude that the action of cyclic stretching also stimulates superior cell proliferation based on our previous pilot study [28]. However, an increase in strain magnitude does not necessarily result in higher differentiation as demonstrated in this study, where 8% strain resulted in the highest tenogenic expression and not at 4% or 12% strain. Yet, based on our previous pilot study [28], hMSCs subjected to 4% strain at 1 Hz provides the best cell proliferation. The choice of strain rate, i.e., 1 Hz in this study was based on our previous study which showed that at this rate the best cellular differentiation in hMSCs was observed [28]. It is worth noting that uniaxial cyclic loading does not result in chondrogenic, adipogenic, or osteogenic differentiation and that, at the prescribed loading regime, cells tend to form distinctive tendon-like cell phenotype. As far as the authors of this paper are aware, these observations have not been previously reported. Another novelty of this study is that specific combinations of strain amounts and rate of tensile loading provide specific hMSC tenogenic differentiation responses as mentioned earlier. It is important to note here that as far as the authors of this paper are aware, there is no consensus on the proper definition of tenogenic differentiation. In trying to ensure that the work done in our study incorporates any characteristics of tenogenic differentiation possible using gene and protein expressions, the work of several studies from different laboratories was used as reference [25, 26, 31, 32]. It is hoped that in doing this, a more global definition of what defines tenogenic differentiation can be made [23].

Our study corroborates previous findings that cell orientation is altered when subjected to cyclic loading [20, 33]. The cell appears to reorientate in a longitudinal axis perpendicular to its original orientation as well as the direction of cyclic loading. This phenomenon appears to be necessary for the reduction of excessive strain that is applied to the cellular structures. In addition, this also results in the increase in specific phenotypic expressions from these cells as previously described [34, 35]. It has been suggested that the mechanisms involved in promoting cellular realignment are dependent on various factors, which includes the rearrangement of intracellular stress fibers due to energy dissipation and the fluctuations in the ionic exchange mechanisms such as the depolarization of voltage-gated channels [36, 37]. Based on our observations, it is likely that the actin stress fibers, which are a major cytoskeletal constituent, may be responsible for the proliferation and differentiation of hMSCs [38, 39]. The AFM and confocal fluorescence microscopic analyses demonstrate these changes occurring in the actin stress fibers which, if based on previous findings, suggest that the change in Young's modulus was ascribable to the development of the cellular cytoskeleton during the differentiation process [40].

Another finding that corroborates previous studies is the fact that hMSCs subjected to tensile cyclic loading result in the apparent increase in the synthesis of collagen type I and type III, and potentially other tenogenic protein expressions [31, 32, 41]. However, whilst our study did not demonstrate any chondrogenic, osteogenic, or adipogenic expressions, these have been reported in others [42–45]. We hypothesized that these differences may be attributable to the different loading types, magnitude, rates, and even the device used to create the mechanical strained environments employed in each of these studies, since it has been shown that different types of mechanical signals will produce different outcomes, i.e., resulting in the differentiation of hMSCs towards a specific lineage [46]. For example, low-amplitude or low-frequency mechanical loading has been shown to promote osteogenic (1 Hz, 3%, 48 h) [31], myogenic (1 Hz, 4%, 24 h) [47], and neuronal (0.5 Hz, 0.5%, 8 h) [15] differentiation of hMSCs. In addition, the action of cyclic compression appears to be a major contributing factor required for MSCs to undergo chondrogenesis [48]. Apparently, loading cells in a uniaxial and biaxial manner will result in different outcomes. In another study using similar rate and magnitudes to ours but employing biaxial loading, MSCs tend to differentiate towards osteogenic lineage [49]. Thus, it is not unexpected that uniaxial cyclic stretch is believed to be of paramount importance in the development of functional musculoskeletal tissues [50] especially for the differentiation of MSCs into tendon/ligament fibroblasts. One aspect that needs to be considered is that the differences observed between our study and that of previous reports [51–53] may have been related to the Flexcell system used in their studies. In contrast to the Strex machine used in our study, this device employs a suction mechanism at the centre of the elastomeric cell culture surface to create the stretching effect. It may be the case that the radial stretching effect of the Flexcell system could have produced compounding compressive forces to the attached cells thus resulting in the osteogenic lineage differentiation. This however remains speculative and would require further supportive findings in future studies. Thus, it can be concluded that different types of mechanical signals will produce different outcomes, i.e., resulting in the differentiation of hMSCs towards a specific lineage [54].

The clinical implication of the study is apparent and may lead to several potential applications. Although further studies are required, it is now possible to extrapolate the data obtained from our study to be applied into patients. In fact, this is not new since many studies have demonstrated that mechanical loading is beneficial to the musculoskeletal system [55]. This particularly applies to the tendon which has been shown to undergo tissue reparative process when subjected to stretching exercises [56, 57]. What is new here in this study is the fact that only a certain combination of strain and cyclic loading rates may be beneficial for multipotent cells such as hMSCs, while other combinations may not be or in fact quite the opposite, may even result in detrimental outcomes. Once the optimal combination has been established, such as that which is observed in the present study, stretching will elicit anabolic responses from the tendon cells. This in turn increases the production of type I collagen in the peritendinous tissues as demonstrated previously [58].

Tendons, being viscoelastic tissues that are stiffer than most other soft tissues, allow the transfer of large tensile forces to occur without causing tissue or cell damage [59]. Indeed, although resistant to tensile forces, tenocytes are still subjected to high mechanical stresses enclaved within a highly mechanoactive environment [60]. However, to study the mechanical processes underpinning the cellular response within an *in vivo* environment would be technically unmanageable; hence, a model such as the one employed in the present study may be more realistic, appropriate, and informative. We recognize the limitations of a system that do not truly mimic the *in vivo* environment; however, these have been considered during our analyses and have not overstated the findings of the present study. We also recognized that although the present study was well designed, several limitations were unavoidable and thus need to be highlighted here. Firstly, it needs to be reminded that as with any *in vitro* studies, the present study does not take into account the complexities of surrounding tissues, and thus, translating the findings into clinical applications would need to be done with caution. Secondly, the present study assumes that the effect of the stretching occurs in a uniform manner, which in reality may not be the case. More so when certain areas within the substrate are subjected to a phenomenon known as differential stretching, as suggested in previous studies [61–64]. Limited by the size of the cell culture flask and the maximal rate of which cells can proliferate, the present study could only be conducted up to 72 hours. There is a downside to this, since it is possible that certain gene expressions such as osteogenic expressions may not have been detected. In previous studies, it appears that culturing MSCs up to 14 days may be needed for these changes to be observed. Hence, it may be the case that the tests from our experiment may have shown false-negative results. It needs to be reminded however that these changes may be better applied for static cultures and probably not applicable to our stretching cultures [65, 66]. Results from other studies seem to suggest that this is the case [53, 67, 68]. Notwithstanding these limitations, the findings of the present study are still valid and useful owing to the robust study design employed. It is however hoped that future studies can be conducted using more advanced techniques that are not subjected to the limitations mentioned above.

## 5. Conclusions

Cells subjected to 1 Hz cyclic uniaxial stretching demonstrated significant maximal tenogenic expression observed but not of other mesenchyme lineages when stretched at 8% strain. No dose-related responses were observed as the result of increased strain magnitude, and it is more likely to be the case that a specific combination of rate and strain magnitude will elicit specific cell responses as demonstrated from our present and previous studies.

## Figures and Tables

**Table 1:** The genes of interest were determined in this study.

Related marker	Gene name	Abbreviation
	Matrix metallopeptidase 3 (stromelysin 1, progelatinase)	*MMP3*
	Proline rich 16	*PRR16*

ECM component	Collagen type I, *α*1	*COL1*
	Collagen type III, *α*1	*COL3*
	Decorin	*DCN*

Tendon lineage	Tenascin C	*TNC*
	Scleraxis homolog A	*SCX*
	Tenomodulin	*TNMD*

Bone lineage	Runt-related transcription factor 2	*RUNX2*
	Alkaline phosphatase, liver/bone, kidney	*ALP*
	Osteocalcin	*OCN*

Cartilage lineage	Collagen type II, *α*1	*COL2*
	Cartilage oligomeric matrix protein	*COMP*
	SRY- (sex-determining region Y-) box 9	*SOX9*

Fat lineage	Peroxisome proliferator-activated receptor, gamma	*PPARG*

Smooth muscle lineage	Transgelin	*TAGLN*

Housekeeping gene	Phosphoglycerate kinase 1	*PGK1*

## Data Availability

The data used to support the findings of this study are included within the article.
